# A Review of the Resistance Mechanisms for *β*-Lactams, Macrolides and Fluoroquinolones among *Streptococcus pneumoniae*

**DOI:** 10.3390/medicina59111927

**Published:** 2023-10-31

**Authors:** Nurul Izzaty Najwa Zahari, Engku Nur Syafirah Engku Abd Rahman, Ahmad Adebayo Irekeola, Naveed Ahmed, Ali A. Rabaan, Jawaher Alotaibi, Shayea A. Alqahtani, Mohammed Y. Halawi, Ibrahim Ateeq Alamri, Mohammed S. Almogbel, Amal H. Alfaraj, Fatimah Al Ibrahim, Manar Almaghaslah, Mohammed Alissa, Chan Yean Yean

**Affiliations:** 1 Department of Medical Microbiology and Parasitology, School of Medical Sciences, Universiti Sains Malaysia, Health Campus, Kubang Kerian 16150, Malaysiaengkunursyafirah@gmail.com (E.N.S.E.A.R.);; 2 Microbiology Unit, Department of Biological Sciences, College of Natural and Applied Sciences, Summit University Offa, Offa PMB 4412, Nigeria; 3 Molecular Diagnostic Laboratory, Johns Hopkins Aramco Healthcare, Dhahran 31311, Saudi Arabia; 4 College of Medicine, Alfaisal University, Riyadh 11533, Saudi Arabia; 5 Department of Public Health and Nutrition, The University of Haripur, Haripur 22610, Pakistan; 6 Infectious Diseases Unit, Department of Medicine, King Faisal Specialist Hospital and Research Center, Riyadh 11564, Saudi Arabia; 7 Medical Laboratory Department, Erhadh Hospital, Dammam 32434, Saudi Arabia; 8 Cytogenetics Department, Dammam Regional Laboratory and Blood Bank, Dammam 31411, Saudi Arabia; 9 Blood Bank Department, Dammam Regional Laboratory and Blood Bank, Dammam 31411, Saudi Arabia; 10 Department of Medical Laboratory Sciences, College of Applied Medical Sciences, University of Hail, Hail 4030, Saudi Arabia; 11 Pediatric Department, Abqaiq General Hospital, First Eastern Health Cluster, Abqaiq 33261, Saudi Arabia; 12 Infectious Disease Division, Department of Internal Medicine, Dammam Medical Complex, Dammam 32245, Saudi Arabia; 13 Department of Medical Laboratory Sciences, College of Applied Medical Sciences, Prince Sattam bin Abdulaziz University, Al-Kharj 11942, Saudi Arabia; 14 Hospital Universiti Sains Malaysia, Universiti Sains Malaysia, Health Campus, Kubang Kerian 16150, Malaysia

**Keywords:** bacterial infections, community-acquired pneumonia (CAP), antimicrobial resistance, fluoroquinolones, AMR

## Abstract

*Streptococcus pneumoniae (S. pneumoniae)* is a bacterial species often associated with the occurrence of community-acquired pneumonia (CAP). CAP refers to a specific kind of pneumonia that occurs in individuals who acquire the infection outside of a healthcare setting. It represents the leading cause of both death and morbidity on a global scale. Moreover, the declaration of *S. pneumoniae* as one of the 12 leading pathogens was made by the World Health Organization (WHO) in 2017. Antibiotics like *β*-lactams, macrolides, and fluoroquinolones are the primary classes of antimicrobial medicines used for the treatment of *S. pneumoniae* infections. Nevertheless, the efficacy of these antibiotics is diminishing as a result of the establishment of resistance in *S. pneumoniae* against these antimicrobial agents. In 2019, the WHO declared that antibiotic resistance was among the top 10 hazards to worldwide health. It is believed that penicillin-binding protein genetic alteration causes *β*-lactam antibiotic resistance. Ribosomal target site alterations and active efflux pumps cause macrolide resistance. Numerous factors, including the accumulation of mutations, enhanced efflux mechanisms, and plasmid gene acquisition, cause fluoroquinolone resistance. Furthermore, despite the advancements in pneumococcal vaccinations and artificial intelligence (AI), it is not feasible for individuals to rely on them indefinitely. The ongoing development of AI for combating antimicrobial resistance necessitates more research and development efforts. A few strategies can be performed to curb this resistance issue, including providing educational initiatives and guidelines, conducting surveillance, and establishing new antibiotics targeting another part of the bacteria. Hence, understanding the resistance mechanism of *S. pneumoniae* may aid researchers in developing a more efficacious antibiotic in future endeavors.

## 1. Introduction

Pneumonia is a respiratory condition characterized by the accumulation of pus and fluid in the alveoli of the lungs [1]. Community-acquired pneumonia (CAP) is a commonly seen subtype of pneumonia. This infection is acquired in non-hospital settings and is a significant global cause of mortality and morbidity. According to the World Health Organization (WHO), lower respiratory tract infections (LRTIs), which include CAP, have been identified as the third leading cause of mortality [2,3]. Over 100 pathogens, including bacteria, viruses, and fungi, are causative agents in the onset of CAP. Nevertheless, *Streptococcus pneumoniae* (*S. pneumoniae*) remains the predominant etiological agent responsible for bacterial CAP [4]. This pneumococcus is also associated with other additional disorders, including bacteremia, otitis media, and meningitis. There exists a correlation between the presence of pneumococcus and death rates seen in cases of bacterial meningitis, with reported values ranging from 16% to 37%. Approximately 30% to 50% of adult survivors continue to have enduring residual symptoms [5,6].

*S. pneumoniae* is a Gram-positive bacterium that exhibits opportunistic behavior by colonizing the mucosal surfaces of the upper respiratory tract (URT) in humans [7]. The presence of the bacteria has been shown to be associated with elevated levels of illness and mortality, especially among those in certain vulnerable populations, such as children below the age of two, individuals with impaired immune systems, and older adults [8]. Despite being a commensal bacterium, *S. pneumoniae* has the potential to induce significant morbidity when it transits from its primary reservoir on mucosal surfaces and disseminates to sterile sites, such as the lungs, leading to the development of pneumonia. Currently, it is concerning that *S. pneumoniae* was frequently identified as the prevalent bacterial co-infection in individuals with coronavirus disease 2019 (COVID-19). The interplay among co-pathogens like *S. pneumoniae*, SARS-CoV-2, and the host poses a significant challenge to the diagnosis, treatment, and prognosis of COVID-19 on a global scale [9]. Moreover, the WHO classified *S. pneumoniae* as one of the twelve bacterial strains that need immediate development of novel therapeutic approaches in 2017 [7].

The primary antibiotic agents used for the eradication of *S. pneumoniae* are *β*-lactams, macrolides, and fluoroquinolones. However, the efficacy of these antimicrobials is diminishing as a result of the establishment of resistance in *S. pneumoniae* against these drugs [10]. According to a 2014 investigation conducted by the WHO on antibiotic resistance, pneumococcus was identified as one of the nine microorganisms that pose a worldwide concern [11]. According to a study conducted by Van Boeckel et al. in 2014 [12], there was a significant rise in global antibiotic use from 2000 to 2010. The usage of antibiotics rose by over 30%, reaching a total of almost 70 billion standard units, up from roughly 50 billion units. The antibiotics commonly used in 2020 were penicillin, macrolides, and cephalosporins. In 2010, the use of antibiotics in India amounted to 13 billion standard units, while China consumed 10 billion and the United States consumed 7 billion standard units. It is worth noting that a standard unit refers to the number of doses sold, as defined by the IMS Health database, which includes pills, capsules, or ampoules [12].

For almost four decades, penicillin G has served as the fundamental therapeutic approach for managing pneumococcal illness. The prevalence of penicillin-resistant pneumococci, first observed in the 1960s, has shown a significant surge in the last decade [13,14,15]. According to a study conducted in 1997, around 33% of pneumococci have shown resistance to penicillin [15]. Regrettably, it has been shown that tetracyclines, clindamycin, chloramphenicol, and trimethoprim-sulfamethoxazole (TMP-SMX) sometimes exhibit little efficacy when used against bacteria that have developed resistance to penicillin [13,14,15]. The capacity of pathogens to evade the lethal effects of certain medications is influenced by patterns of antibiotic use [16]. The high prevalence of pneumococcus resistance to *β*-lactams and macrolides in Southern Europe’s nations is a matter of great worry, with estimates suggesting that it may surpass 20% [17,18].

Most penicillin-sensitive and penicillin-resistant *S. pneumoniae* are vulnerable to rifampicin, fluoroquinolones, and carbapenems and have been extensively studied and documented in previous research [14,15,16,19]. The susceptibility of the majority of strains to carbapenems exceeds 90% [15]. However, it has been observed that there have been 12 instances where pneumococcus has shown a loss of sensitivity to fluoroquinolones, despite the fact that current fluoroquinolones have demonstrated effectiveness against over 99% of isolates [15]. Vancomycin continues to exhibit efficacy against all strains of pneumococci. However, several strains of *S. pneumoniae* have shown a degree of tolerance towards vancomycin, hence indicating the potential emergence of vancomycin resistance in the near future [14,15].

The management of pneumococcus poses challenges due to many factors, including the extensive use of antibiotics, the emergence of several resistant strains, the phenomena of serotype replacement and capsular remodeling, and the horizontal transfer of genes associated with antibiotic resistance [8]. This review provides insight into the resistance of *S. pneumoniae* to anti-pneumococcal drugs.

## 2. Etiology

*Streptococcus pneumoniae* exhibits facultative anaerobic characteristics. It has the ability to display either alpha-hemolytic or beta-hemolytic behavior, depending on whether it is exposed to aerobic or anaerobic circumstances, respectively. This bacterium is classified under the *Streptococcus* genus. Additional bacterial species that have been detected in CAP include *Staphylococcus aureus*, *Hemophilus influenzae*, *Pseudomonas aeruginosa*, *Klebsiella pneumoniae*, and anaerobic bacteria. It is noteworthy to observe that the prevalence of *S. pneumoniae* seems to be progressively declining as a result of the growing acceptance and use of pneumococcal vaccinations. Atypical infections, such as *Chlamydophila pneumoniae* and *Mycoplasma pneumoniae*, are gradually becoming significant pathogens, particularly among the younger adult population [20].

Several risk factors have been found in individuals with CAP. These risk factors include immunosuppression, heavy alcohol use, advanced age (over 70 years), asthma, and prolonged exposure to overcrowded settings, among others [21,22].

## 3. Pathogenesis and Immunopathogenesis of Pneumonia

**Figure 1:**
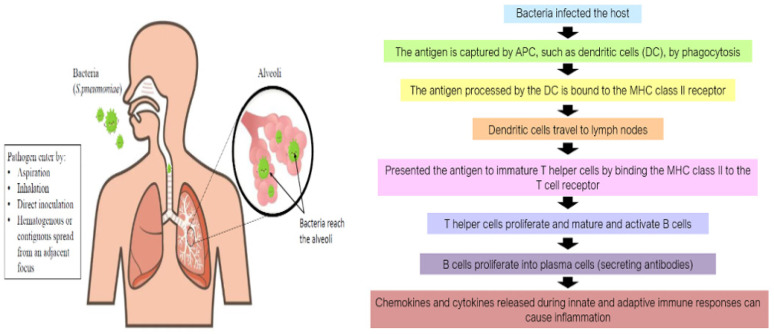
Pathogenesis and immunopathogenesis of pneumonia by *S. pneumoniae*. The host can become infected by the pathogen in a number of ways, such as through aspiration, direct inoculation, breathing in, and hematogenous or contiguous spread from a nearby focus. Once the pathogen reaches the alveoli, it multiplies and evokes a host response.

The onset of bacterial pneumonia often starts with the introduction of a causative bacterium, such as *S. pneumoniae*, into the respiratory system of a host (Figure 1). The pathogen infiltrates the alveoli, undergoes replication, and elicits host immune responses. The pathogen may obtain access to the lower respiratory tract of the host by several means, including aspiration, inhalation, direct inoculation, and hematogenous or contiguous transmission from a nearby source. While direct inoculation resulting from a penetrating thoracic injury or contiguous spread from an infection site, such as mediastinitis, is theoretically feasible, it is generally uncommon [23]. Hematogenous dissemination may develop in individuals with tricuspid endocarditis who engage in intravenous drug misuse. However, the primary possible pathways consist of the aspiration of a small volume of bacterial pathogens into the host’s oropharynx, which may occur during sleep, as well as the inhalation of contaminated droplets. Microaspiration is a phenomenon that may occur with notable frequency even among individuals who are considered to be in good health. However, the development of pneumonia as a consequence of microaspiration is quite rare. The incidence of pneumonia is primarily influenced by factors such as the amount of aspirated material, the concentration of pathogenic bacteria, the frequency of aspiration events, and the virulence of the aspirated bacteria in relation to the host’s immune system [2]. In addition to technical safeguards, it is crucial to have both innate and adaptive host defenses in place to provide protection against such occurrences [24].

The initial stage of the *S. pneumoniae* life cycle in the host involves nasopharyngeal colonization. The bacteria employ various factors, such as polysaccharide capsules, peptidoglycan-N-acetylglucosamine deacetylase (PgdA), exoglycosidases-neuraminidase A (NanA), pneumococcal adherence, and virulence proteins, to facilitate their colonization [25]. The first mechanisms used to counteract the invasion of pathogens include the presence of nose hairs and turbinates, as well as the functional reflexes of coughing and gagging. Additionally, the tracheobronchial tree, characterized by its branching structure, plays a crucial role in effectively clearing mucus by the action of cilia. This information is supported by reference [23]. *S. pneumoniae* uses the matrix metalloprotease ZmpA to cleave mucosal IgA to avoid complement activation and clearance by the mucociliary flow [26]. Pneumonia develops when *S. pneumoniae* moves from the nasopharyngeal site to the lungs’ alveoli [25]. The alveoli serve as a barrier against pathogens, which are effectively countered by surfactant proteins and alveolar macrophages.

The macrophages and dendritic cells (DCs) serve as the primary immune defense against the bacteria. The antigen is captured by the DCs by phagocytosis. DCs then travel to lymph nodes, where immature T and B cells are situated. The antigen processed by the DCs is then bound to the MHC class II receptor and presented to immature T cells by binding the MHC class II to the T cell receptor. T cells proliferate, mature, and activate B cells, which in turn proliferate into antibody-secreting plasma cells [27]. In the event that these defensive mechanisms prove ineffective, allowing the pathogen to remain and proliferate, the host will initiate inflammatory responses that give rise to a multitude of the signs and symptoms often recognized in patients with pneumonia. The overproduction of proinflammatory cytokines released during innate and adaptive immune response can cause inflammation and may exacerbate the pathological condition, leading to sepsis, shock, organ dysfunction, or potentially fatal outcomes. It is believed that the presence of inflammatory mediators, including tumor necrosis factor (TNF), interleukin-1 (IL-1), interleukin-8 (IL-8), and granulocyte-colony stimulating factor, contributes to the development of fever and the release and recruitment of neutrophils to the lung [23]. Leakage of fluid into the capillaries of the alveoli may occur, resulting in the accumulation of fluid in the alveoli and potentially causing a decrease in oxygen levels in the blood (hypoxemia). Patients may have fatal outcomes due to alterations in lung capacity and compliance in extreme circumstances [28].

In general, the development of pneumonia occurs in patients as a result of several phases of tissue alterations. During the first phase, the development of edema is seen as a consequence of proteinaceous exudate in the alveoli. This is then followed by a stage known as red hepatization, characterized by the buildup of red blood cells. Following is the subsequent phase known as gray hepatization, which is distinguished by the process of red cell lysis and disintegration, accompanied by the deposition of fibrin and neutrophils. The subsequent step is characterized by the resolution process, which encompasses the activities of macrophages, the removal of waste material, and the reduction in inflammatory reactions [23].

## 4. Overview of Antibiotic

Antibiotics are pharmaceutical substances that aid in the prevention of infections caused by bacteria. In order to achieve this objective, one must either eliminate the bacteria or inhibit their replication or reproduction. The term “antibiotic” often denotes a substance that is antagonistic to living organisms [29]. A pharmaceutical compound is categorized as an antibiotic if it has the ability to eradicate bacterial microorganisms residing inside the human body. During the 1920s, the development of the first antibiotics coincided with the observation that strep throat, a common bacterial infection, was a contributing factor to death rates [30]. Moreover, the practice of surgery posed significant risks. Nevertheless, subsequent to the breakthrough of antibiotics in the 1940s, humans were able to endure formerly lethal diseases, extend their lifespan, and undertake more secure medical interventions. The vast majority of microorganisms residing inside the human body are considered to be harmless or nonpathogenic. Certain bacteria may play a role in many physiological processes inside the human body. However, it is important to note that almost every organ has the potential to be infected by microbes. Antibiotics have the capability to just address ailments of bacterial origin, hence rendering them ineffective in combating disorders stemming from viral etiology. Nevertheless, there are instances where distinguishing between a bacterial or viral disease might provide challenges. Certain antibiotics possess the ability to effectively combat a wide range of bacterial strains, therefore earning them the designation of broad-spectrum antibiotics. Certain microbes, referred to as narrow-spectrum [31], are exclusively targeted by others.

## 5. Antibiotic Resistance and Its Effects

When antibiotics are provided in a suitable and accurate manner, they may effectively combat bacterial infections [31]. Nevertheless, it has been shown that a significant proportion, around 50%, of antibiotic use is deemed unnecessary. One of the contributing factors to the development of antibiotic resistance is the overuse of antibiotics. Over the course of time, bacteria undergo evolutionary processes that lead to the emergence of highly resistant strains, sometimes referred to as super bacteria or superbugs. They are modified to such an extent that antibiotics lose their efficacy. Due to the absence of pharmaceutical interventions capable of eradicating them, these entities provide a significant reason for worry. One effective approach to mitigating the proliferation of antibiotic-resistant bacteria is the judicious and appropriate use of antibiotics [30].

Antibiotics are used to prevent and treat bacterial illnesses. Resistance to antibiotics arises when bacteria have adapted to the presence and effects of these antimicrobial agents. Neither people nor animals develop antibiotic resistance; rather, it is only bacteria that gain this resistance. The aforementioned bacteria have the capability to cause infections in both people and animals. It is worth noting that the treatment of diseases caused by these bacteria poses more challenges compared to those caused by non-resistant strains [32]. The phenomenon of antibiotic resistance has been shown to be associated with elevated mortality rates, prolonged hospitalization periods, and escalated healthcare expenditures. Hence, there is an urgent need for the fast reformation of the prescription and use of antibiotics on a worldwide scale. Despite the advancements in pharmaceutical research and the discovery of novel drugs, the issue of antibiotic resistance continues to pose a substantial and enduring global menace. Furthermore, in order to mitigate the transmission of illnesses, it is important to focus on modifying behaviors that promote improved food hygiene, practicing safer sexual practices, maintaining proper hand hygiene, and obtaining necessary vaccines [30,33].

The prevalence of antibiotic resistance is escalating to alarmingly high levels on a global scale. Antimicrobial resistance (AMR) might cost USD 300 billion to USD 1 trillion globally by 2050. The global proliferation of novel resistance mechanisms poses a significant challenge to effectively treating prevalent infectious diseases. The diminishing efficacy of antibiotics has resulted in increased challenges in the treatment of several illnesses, including gonorrhea, TB, septicemia, and pneumonia, sometimes rendering them resistant to available medical interventions [29,34]. Furthermore, the availability of over-the-counter medications for both human and animal consumption accelerates the emergence and dissemination of antibiotic resistance. In nations without standardized treatment guidelines, both medical practitioners and veterinarians regularly engage in the excessive prescription of antibiotics, while the general populace tends to exhibit a pattern of excessive use. In the absence of prompt intervention, the present circumstances may lead to a future characterized by a post-antibiotic era, whereby commonplace ailments and small injuries may regain their potential to result in fatality [35,36].

## 6. Prevalence of Antimicrobial Resistance in *S. pneumoniae*

The implementation of pneumococcal conjugate vaccines (PCV) PCV7 and PCV13 has successfully decreased invasive pneumococcal infections and provided herd protection for non-vaccinated people. However, the burden of pneumococcal infections remains high due to the limited access to vaccines in developing countries and the limited serotype coverage provided by the established vaccines. It has been noted that antibiotic resistance increased in the post-PCV era [37,38]. *S. pneumoniae*, a bacterium well recognized as a significant contributor to CAP on a worldwide scale, regrettably exhibits resistance to several medications. The emergence of antibiotic resistance in *S. pneumoniae* poses a significant concern to healthcare systems globally as it results in treatment failure. The consequences of this resistance issue include elevated mortality rates, prolonged morbidity periods, and higher costs of medical care [39,40]. The early documentation of *S. pneumoniae’s* resistance to penicillin, followed by its subsequent resistance to other categories of antibiotics, has posed challenges in selecting appropriate antibiotic treatments. The susceptibility of *S. pneumoniae* to *β*-lactams and macrolides has shown a gradual decline [41]. The prevalence of resistance to fluoroquinolones, tetracycline, and trimethoprim-sulfamethoxazole (TMP-SMX) is also being progressively reported [10].

According to Cherazard et al. 2017 [10], the study conducted in the United States revealed varying prevalence rates of *β*-lactam resistance in *S. pneumoniae*, ranging from less than 1% to 41.8%, depending on the specific *β*-lactam medicines. The percentage range of penicillin is seen to be between 13.8% to 41.8%. The percentage distribution of cephalosporins is as follows: cefuroxime accounts for 29.9%, ceftriaxone for 11.7%, ceftaroline for 0–1%, and imipenem for 23.8%. In addition, it has been shown that the prevalence of macrolide resistance ranges from 20% to 40%. In contrast to the resistance shown in *S. pneumoniae* to the other two antimicrobials, the rate of fluoroquinolone resistance ranged from less than 1% to 2%, which was the lowest (Table 1).

Based on a report acquired in the United States, it was shown that the prevalence of resistance to *β*-lactams exhibited the greatest rate, followed by resistance to macrolides, and subsequently fluoroquinolones. The resistance mechanisms pertaining to each medicine are enumerated above.

## 7. Action and Resistance Mechanisms for *β*-Lactams, Macrolides, and Fluoroquinolones among *S. pneumoniae*

### 7.1. β-Lactams

The structural foundation of *β*-lactam antibiotics consists of a beta-lactam ring, which is a four-membered ring containing nitrogen. The D-Ala-D-Ala peptide sequence, which serves as the substrate for cell wall transpeptidases, has a structural conformation akin to that of a ring. Currently, there are four primary categories of *β*-lactam compounds [42]. The mechanism of action of these drugs involves the specific targeting and suppression of cell wall production by their attachment to the enzymes involved. It is important to note that the beta-lactam ring plays a crucial role in their mode of action. Penicillin-binding proteins (PBPs) refer to a group of enzymes that are affixed to the cellular membrane. The PBPs are categorized into 4–6 distinct categories according to the specific bacterial species they target. The transpeptidases, also known as PBPs, play a vital role in the cross-linking of the cell wall, which is essential for the organism’s survival [43,44].

**Figure 2:**
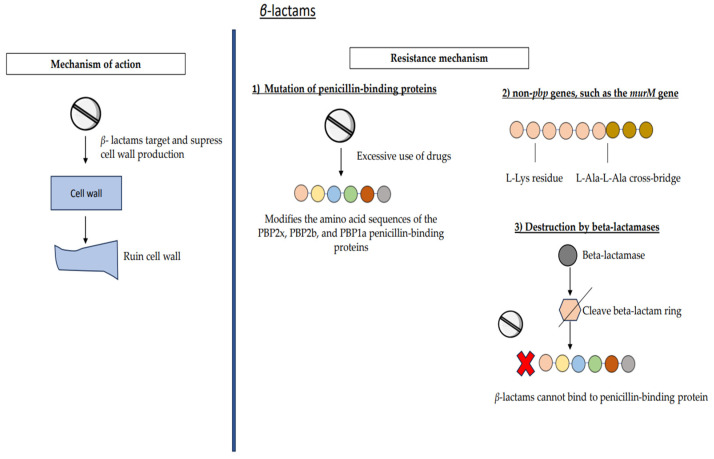
Mechanism of action and resistance mechanism of *β*-lactams. There are few resistance mechanisms that involve mutation of penicillin-binding proteins, non-pbp genes, and destruction by beta-lactamase.

The D-Ala-D-Ala peptide terminus serves as the inherent substrate for the transpeptidase activity involved in the production of peptidoglycan in the cell wall. This peptide terminus has a three-dimensional conformation that is replicated by the four-membered ring structure seen in *β*-lactam antibiotics. *β*-lactam medications inhibit the synthesis of cell walls by forming strong bonds with the active site of transpeptidase (Figure 2) [45]. Cell death occurs as a result of osmotic instability caused by inadequate cell wall synthesis, or the binding of *β*-lactam to PBP may initiate a series of reactions that ultimately result in autolysis and cell demise. *β*-lactam antibiotics have efficacy against both Gram-positive and Gram-negative bacteria. Nevertheless, the effectiveness of these antibiotics differs as a result of disparities in the cellular structures of the two bacterial classifications (e.g., Gram-negative bacteria possess an outer membrane, whereas Gram-positive bacteria lack one) [42,46].

*β*-lactam antibiotics, such as penicillin, cephalosporins, and carbapenems, were formerly considered very effective antimicrobial agents for the treatment of individuals infected with *S. pneumoniae* subsequent to its identification in 1928 by Alexander Fleming. Previously, it functioned as an antimicrobial agent with a high susceptibility to *S. pneumoniae*. Nevertheless, it was in 1967 that the first instance of penicillin resistance in *S. pneumoniae* was documented in Australia [47]. The minimum inhibitory concentrations (MICs) of *β*-lactams have shown an upward trend over time, and a considerable number of studies have been published on the emergence of *β*-lactam-intermediate and *β*-lactam-resistant strains of *S. pneumoniae*. The aforementioned situation has resulted in a decline in the use of *β*-lactams as a viable therapeutic alternative [48].

**Figure 3:**
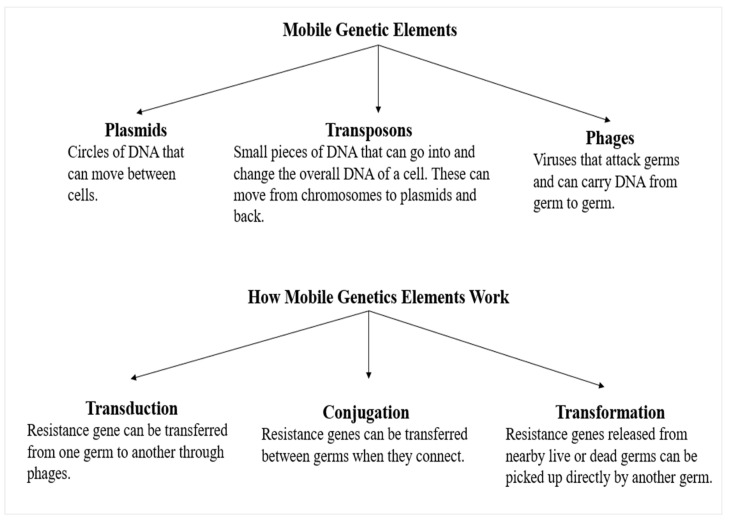
How antibiotic resistance moves directly from germ to germ. Resistance traits are transmissible from generation to generation. They can be transmitted directly between bacteria via mobile genetic components like plasmids, transposons, and phages. The genetic component works by transduction, conjugation, and transformation.

**Figure 4:**
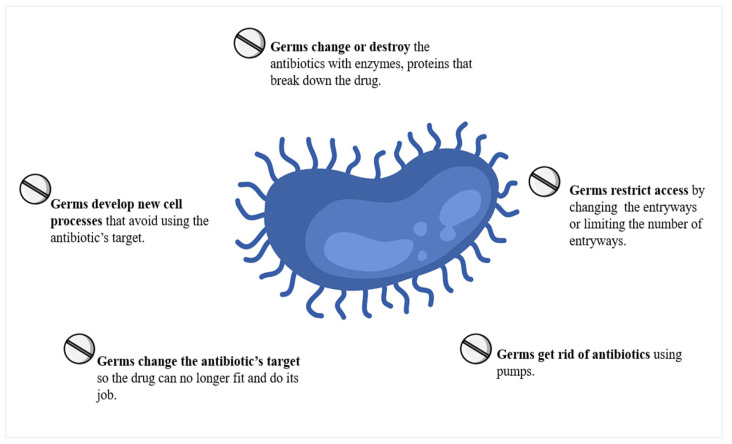
How bacteria fight back against antimicrobials. Antibiotics combat bacteria. However, bacteria adapt and discover new ways to survive. Their defensive strategies are known as resistance mechanisms.

There exists a notion suggesting that the development of resistance in pneumococcus and commensal streptococcus may be attributed to a minimum of two distinct phases. The process involves the selection of commensal streptococci that are resistant to mutations in their *pbp* genes, followed by the transfer of these resistant genes to competent pneumococci by homologous recombination. This information is summarized in Table 1 and shown in Figure 2, Figure 3 and Figure 4 [49]. The mutations progressively accumulate inside three transpeptidases that play a crucial role in the manufacture of the cell wall, specifically known as penicillin-binding proteins (PBPs). Excessive use of the medication has resulted in a genetic alteration that modifies the amino acid sequences of the PBP2x, PBP2b, and PBP1a penicillin-binding proteins (Figure 3) [50]. The presence of even minor and diverse fragments arising from mutations may significantly impact the modification of *pbp* genes, leading to the development of *β*-lactam resistance in commensal streptococcus and *S. pneumoniae*. The modified bacteria exhibit a competitive edge in the presence of antibiotics as a result of these genetic alterations, which decrease the binding affinity of transpeptidases for the drug while preserving the enzyme’s functioning [51].

The *pbp* genes in resistant pneumococcus strains have a mosaic pattern in contrast to the same sections of susceptible pneumococcus. These mosaic patterns consist of sequence blocks of varying lengths, which may diverge by up to 20% and 10% at the DNA level, respectively [51]. The mosaic structure seen in this study may be attributed to the transfer of genes between species originating from *Streptococcus mitis* and *Streptococcus oralis*, which are believed to be potential donors. These two species coexist in the nasopharynx, which serves as their shared biological niche. The integration of exogenous DNA from *β*-lactam-resistant Streptococcus strains that inhabit the same ecological niche is shown in Figure 4 [49,52].

Furthermore, it has been hypothesized that non-*pbp* genes, such as the *murM* gene, may be associated with the resistance of *S. pneumoniae* to *β*-lactam antibiotics. The operon under consideration is responsible for encoding transferases that facilitate the elongation of the peptidoglycan stem by adding an L-Ala-L-Ala cross-bridge to the L-Lys residue. Nevertheless, there is a lack of confidence about the precise mechanism by which these elongations contribute to *β*-lactam resistance [53,54]. A further mechanism of resistance involves the enzymatic degradation of *β*-lactam antibiotics by bacteria via the production of beta-lactamase enzymes [55]. The loss of the medication’s capacity to bind to PBPs and inhibit cell wall construction occurs when beta-lactamases cleave the beta-lactam ring (Figure 2). Nevertheless, it is important to note that not all *β*-lactams possess the ability to undergo hydrolysis by all beta-lactamases. For example, the staphylococcal beta-lactamase enzyme exhibits rapid hydrolysis of penicillin and its derivatives. However, it does not possess the ability to hydrolyze other cephalosporins, such as imipenem [42,43].

### 7.2. Macrolides

**Figure 5:**
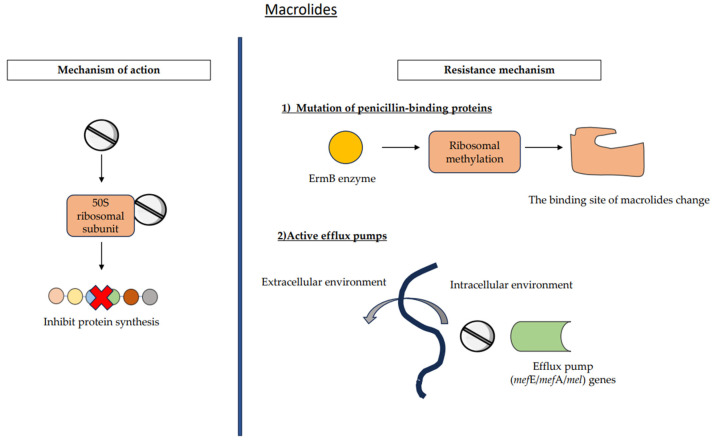
Mechanism of action and resistance mechanism of macrolides. Mutations of penicillin-binding proteins and active efflux pumps are involved in the resistance mechanisms of macrolides among *S. pneumoniae*.

Following the emergence of penicillin resistance in *pneumococcus*, macrolides have emerged as an alternative therapeutic option for treating pneumococcal infections. Macrolides, including erythromycin, azithromycin, and clarithromycin, are a class of bacteriostatic antibiotics that exert their antimicrobial effects by binding to the 50S ribosomal subunit, therefore inhibiting protein synthesis (Figure 5) [56,57]. The primary origin of macrolides may be attributed to *Saccharopolyspora erythraea*, a bacteria found in soil that is also referred to as *Streptomyces erythreus*. The efficacy of macrolides against the majority of Gram-negative bacterial species, excluding enterococci, is limited due to challenges associated with absorption resulting from Gram-negative outer membranes. In addition, they have shown efficacy against many bacterial species, including *Legionella*, *Campylobacter*, *Mycoplasma*, *Treponema*, *Bordetella*, *Chlamydia*, *Chlamydophila*, and *Borreli*, as indicated by sources [35,58].

The 23S ribosomal RNA molecule, among other ribosomal proteins, serves as a particular target for the macrolides’ attachment to the 50S ribosomal subunit [59]. Macrolides have inhibitory effects on bacterial protein synthesis, but via distinct mechanisms that target different phases of the process. The process of peptidyl transfer is hindered by molecules consisting of 16 members, whereas the movement of peptidyl-tRNA is impeded by macrolides composed of 14 members. Based on the prevailing idea, it is posited that macrolides have an inhibitory effect on protein synthesis by inducing the dissociation of peptidyl-tRNA from ribosomes in the elongation phase [60,61].

The increased prevalence of macrolide resistance in *S. pneumoniae* is associated with the widespread use of macrolide antibiotics. The resistance of pneumococcus to macrolides is thought to be primarily facilitated by two processes. One method involves the modification of the ribosome via an enzyme carried by the erythromycin-resistant methylase (*erm*B) gene. The other mechanism involves the presence of active efflux pumps expressed by macrolide efflux (*mef*E/*mef*A/mel) genes, as shown in Table 1 and depicted in Figure 4 [8].

#### 7.2.1. Ribosomal Alteration

The ribosomal methylase of *S. pneumoniae* is primarily encoded by *erm*B, which produces a gene product responsible for the demethylation of the target site on the 23S rRNA. The gene in question is the most often observed predictor of macrolide resistance in *S. pneumoniae* [62]. The process of ribosomal methylation, facilitated by the ErmB enzyme, confers resistance to macrolide, lincosamide, and streptogramin B (MLS_B_ phenotype). Furthermore, it has been shown that the phenotype plays a crucial role in conferring a substantial degree of resistance to macrolides. The modification of the binding site for macrolide and lincosamide via the presence of a specific variation in the ermB gene results in the acquisition of full resistance to clindamycin in some cases [63].

The presence of inducers such as erythromycin allows for the translation of ErmB at a high level, as shown by previous research [64]. The upstream regulatory region of the *erm*B gene in pneumococcus plays a crucial role in determining the mode of *erm*B production, either inducible or constitutively generated at high levels. The regulatory gene consists of many components, including a brief leader peptide called *erm*BL, which contains its ribosome binding site (RBS1). Additionally, it has a non-translational loop-stream structure, followed by certain coding sequences of *erm*B (ermB’). Finally, it includes another RBS2 [62,65]. Moreover, the presence of *erm*BL2, an extra leader peptide, is essential for the stimulation of gene expression mediated by erythromycin [66]. When the expression of *erm*B is capable of being induced, it is hypothesized that the stalling of ribosomes and the stability of mRNA play a role in regulating its expression [67,68,69]. Erythromycin-induced ribosome stalling at the eleventh codon (Asp) of *erm*BL leads to a conformational change in the mRNA, facilitating the accessibility of the *erm*B-RBS2 for the translation of *erm*B [65].

Translational attenuation leads to the repression of erm gene expression in the absence of an inducing agent [62]. The ribosome binding site 2 (GGAG) and the AUG start codon of the *erm*B mRNA were masked by a stem-loop structure. In the presence of erythromycin, a distinct stem-loop structure undergoes modification, resulting in the exposure of the RBS2 and start codon sequences of the *erm*B gene, therefore initiating the production of *erm*B [65].

#### 7.2.2. Active Efflux Pumps

The phenomenon of antibiotic efflux involves the active transport of medicines from the intracellular environment to the extracellular environment via the use of efflux pumps, as seen in Figure 2 [70]. Occasionally, heightened quantities of antibiotics may surpass this mechanism; nevertheless, the incidence of resistance is on the rise [10]. The macrolide efflux (*mef*E/*mef*A/*mel*) genes are responsible for encoding efflux pumps in pneumococcus (Figure 5) [8,62]. The *mef*A gene is responsible for encoding the resistance mechanism. Historically, this often resulted in a minimal degree of resistance [10].

The *mef*E/*mel* operon encodes the mechanism of pneumococcal macrolide efflux, which occurs via a process that remains incompletely elucidated [71]. In order for *S. pneumoniae* to develop resistance to macrolides, the presence of both *mef*E and *mel* genes is required. Both genes are situated on the macrolide efflux genetic assembly (mega) element. The expression of the gene is regulated by a promoter that is activated by macrolide antibiotics with 14- and 15-membered rings, such as clarithromycin, azithromycin, and erythromycin [62,72].

The *mef*E gene in *S. pneumoniae* is responsible for encoding a protein consisting of 405 amino acids. This protein belongs to the major facilitator superfamily and functions as an efflux pump, using the energy derived from the proton motive force to expel molecules from the cells. *Mel*, also known as *msr*D, is a homologous counterpart of the *S. aureus* gene *mrs*A. The genetic sequence encodes for a protein belonging to the ATP-binding cassette (ABC) transporter family, although it does not possess the typical hydrophobic and membrane-binding domains often seen in such proteins. According to previous research [62], it is anticipated that Mel would engage in interactions with transmembrane complexes that are encoded by chromosomes.

The combination of *Mef*E and *mel* has a synergistic effect, resulting in the development of bacterial resistance to macrolides. Additionally, they serve as the constituents of the efflux pump in *S. pneumoniae.* The mechanism of macrolide efflux in *S. pneumoniae* involves the transportation of macrolides from ribosomes by the *mel* protein, which then displaces the macrolide molecules to *mef*E for efflux [62,73]. In addition, the *mef*E/*mel* gene promotes the development of resistance to the human antimicrobial peptide LL-37 by stimulating the production of efflux pumps. The results suggest that the efflux pump is activated during nasopharyngeal colonization [74].

### 7.3. Fluoroquinolones

**Figure 6:**
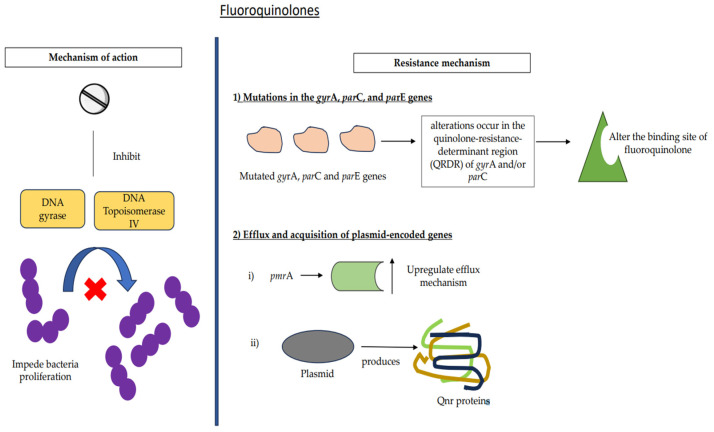
Mechanisms of action and resistance for fluoroquinolones. Mutations in the *gyr*A, *par*C, and *par*E genes, as well as efflux and acquisition of plasmid-encoded genes, are involved in the resistance mechanisms of fluoroquinolone among *S. pneumoniae*.

Fluoroquinolones are potent antibacterial agents that specifically inhibit the activity of DNA gyrase and DNA topoisomerase IV enzymes [36,75]. Gyrase is responsible for the introduction of negative super-coils into DNA, which serves to alleviate the torsional stress that is believed to build up before the formation of transcription and replication complexes [76]. Topoisomerase IV has substantial decatenation activity. The inhibition of gyrase and topoisomerase IV, which are essential enzymes, is expected to impede bacterial proliferation (Figure 6). In contrast, fluoroquinolones exert a detrimental effect on cellular function by forming drug/enzyme/DNA complexes that encapsulate the enzymes responsible for DNA replication. These complexes are stabilized by proteins, therefore facilitating the repair of double-stranded DNA breaks [77,78].

DNA gyrase is responsible for introducing negative superhelical twists to the double helix structure of bacterial DNA before the initiation of replication, with the purpose of facilitating the efficient separation of daughter chromosomes [79]. The aforementioned mechanism plays a crucial role in the commencement of DNA replication as it facilitates the binding of initiation proteins. The DNA gyrase, which is composed of two monomeric GyrA and two monomeric GyrB subunits, is encoded by the *Gyr*A and *Gyr*B genes, respectively [80]. The process of segregating into two daughter cells at the conclusion of a replication cycle is facilitated by decatenation, which involves the removal of interlinking between daughter chromosomes. This decatenation process is executed by an enzyme called topoisomerase IV. Topoisomerase IV consists of a dimeric assembly including two *Par*C subunits (known as *Grl*A in the context of *Staphylococcus aureus*) and two *Par*E subunits (referred to as *Grl*B in the context of *Staphylococcus aureus*). The production of these entities is facilitated by the *Par*C and *Par*E genes, as stated in reference [81].

The resistance of pneumococcus to fluoroquinolones is comparatively lower when compared to the other two agents. This is mostly attributed to the restricted use of this antibiotic owing to its association with the development of articular cartilage injury in weight-bearing joints in animal models [82,83]. *S. pneumoniae* acquires resistance to fluoroquinolones via three main mechanisms: the accumulation of mutations in its genome, the increased efflux of the drug, and the acquisition of plasmid-encoded genes (refer to Table 1 and Figure 2 and Figure 4) [84].

#### 7.3.1. Mutations

The genes *gyr*A and *gyr*B are responsible for encoding DNA gyrase, while *par*C and *par*E are responsible for encoding topoisomerase IV [85]. Mutations in the *gyr*A, *par*C, and *pa*rE genes may occur spontaneously or gradually [84,86]. As a consequence, alterations occur in the quinolone-resistance-determinant region (QRDR) of *gyr*A and/or *par*C, leading to modifications in the fluoroquinolone binding site and a reduction in the affinity of the drug for the enzyme–DNA complex (Figure 6). The development of resistance to ciprofloxacin is exclusively attributed to mutations that arise specifically at the *par*C gene. Nevertheless, the development of resistance to the most recent fluoroquinolones has been shown to be associated with *par*C and *gyr*A mutations [10,84,86].

#### 7.3.2. Efflux and Acquisition of Plasmid-Encoded Genes

*S. pneumoniae* may develop resistance to fluoroquinolones via the upregulation of the efflux mechanism, which is attributed to alterations in regulatory genes [87]. According to existing literature, there is a prevailing belief that the regulatory gene known as *pmr*A plays a significant role in regulating the production of efflux pumps. However, it is worth noting that the genes *pat*A and *pat*B have also been associated with the emergence of resistance to fluoroquinolone antibiotics [86,87].

The phenomenon of plasma-mediated resistance to quinolones occurs via the production of Qnr proteins by plasmids [10]. Nevertheless, the precise mechanism behind the transferable resistance and the incidence of fluoroquinolone-resistant plasmids in clinical environments remain unclear [88].

## 8. Current Trends versus New Trends in Terms of Diagnostics

At present, the diagnosis of AMR is commonly conducted by two established methods: whole genome sequencing for antimicrobial susceptibility testing (WGS-AST) and direct AST. Regrettably, the conventional method of assessing levels of antibiotic resistance, known as AST, has proven to be ineffective and has failed to provide an understanding of the mechanisms behind AMR [32,89]. The WGS-AST method provides a quick, dependable, and precise diagnostic tool for AMR. However, the effective extraction of data requires the use of extensive and complex datasets with large dimensions. Consequently, the use of AI technology has led to the implementation of the subsequent methodologies [90,91].

## 9. Role of Artificial Intelligence in Overcoming AMR

In recent times, there has been significant evidence showcasing the commendable efficacy of artificial intelligence (AI) in the management and control of AMR. AMR AI solutions play a role in improving doctors’ prescriptions and easing their work. One instance of the use of sequencing-based AI applications is the investigation of AMR [92]. Furthermore, the acquisition of clinical data from available health records in AI systems serves to develop clinical decision support systems that might potentially aid physicians in monitoring trends related to AMR and promoting the judicious use of antibiotics. In addition, the utilization of AI applications is prevalent in the field of synergistic drug combination studies and the advancement of innovative antibiotics [93]. Random forests (RF), naïve Bayes (NB), decision trees (DT), support vector machines (SVM), and artificial neural networks (ANN) are among the most commonly used AI approaches for AMR [32,94].

Despite the increasing significance of AI technology as a means for AMR predictions, more efforts are required to further this field. While the use of the IR-spectrometer approach or AI-based FAST has the potential to enhance the efficiency of AST, it should be noted that the workflows associated with these methods may be too complex for individuals without professional expertise to effectively implement [89]. In addition, the development of a comprehensive WGS-AST model for numerous species using a limited training dataset necessitates the creation of a substantial database that can support advanced artificial intelligence techniques such as transfer learning and other methodologies [93]. In order to effectively tune its crucial parameters and be applicable to specific species, the WGS-AST model requires a substantial training dataset. Consequently, this will become imperative for future endeavors.

## 10. Strategies to Curb the Resistance Issue

Currently, the use of antibiotics is the only method accessible for treating pneumococcal diseases [95]. Without intervention, it is predicted that AMR will cause 10 million deaths annually by 2050 [96]. Developing a new antibiotic that targets MurJ might help combat bacterial resistance. MurJ is important in peptidoglycan biogenesis because it acts as the flippase of Lipid II across the cytoplasmic membrane [97,98]. Peptidoglycan is the cell wall layer that protects bacteria from being lysed. In order to synthesize the peptidoglycan, MurJ must flip Lipid II across the membrane [99]. Therefore, a new antibiotic targeting the MurJ will disturb the peptidoglycan synthesis, hence making it unable to protect bacteria from being lysed. Another strategy to curb the resistance issue is by decreasing antibiotic usage. This can be performed via educational initiatives and through guidelines for healthcare workers. Furthermore, ongoing surveillance is also necessary in order to identify the presence of resistance in new strains or increase the rate of resistance in current bacterial strains [8].

## 11. Conclusions

*Streptococcus pneumoniae* is a bacterial pathogen that has persistent mechanisms to avoid eradication. Fluoroquinolones had the lowest rates of *S. pneumoniae* resistance emergence among the three major antibacterial agents. *S. pneumoniae* has evolved many methods to counteract the action of antimicrobials such as *β*-lactams, macrolides, and fluoroquinolones. It is hypothesized that a genetic modification in penicillin-binding proteins is responsible for the phenotypic expression of resistance to *β*-lactam antibiotics. Two mechanisms of macrolide resistance are alterations in the ribosomal target site and the presence of active efflux pumps. In the context of fluoroquinolone resistance, it is seen that many factors contribute to this phenomenon, including the accumulation of mutations in the bacterial genome, enhanced efflux mechanisms, and the acquisition of genes encoded by plasmids. Despite the growth of AI technology in the field of AMR, there remains a significant amount of work that must be undertaken in order to further advance this domain. Hence, the development of a novel and effective therapeutic intervention is essential to combating the bacterial infection.

## Figures and Tables

**Table 1:** The prevalence of antimicrobial resistance in *S. pneumoniae* and the process behind its development.

Antimicrobial Classes	Resistance Mechanism	Resistance Prevalence	
Beta-lactams	(1) Mutation of penicillin-binding proteins (2) Non-*pbp* genes, *murM* gene (3) Destruction by beta-lactamases	Penicillin	Penicillin G: 13.8%
			Penicillin V: 41.8%
		Cephalosporins: <1–29.9%	
		Cefuroxime: 29.9%	
		Ceftriaxone: 11.7%	
		Ceftraroline: 0–<1%	
Macrolides	(1) Ribosomal alteration (2) Active efflux pumps	20–40%	
Fluoroquinolones	(1) Mutations in *gyrA*, *parC*, and *parE* regions (2) Efflux pumps (3) Acquisition of plasmid-encoded genes	<1–2%	

## Data Availability

Not applicable.
